# Using Mobile Health (mHealth) and Geospatial Mapping Technology in a Mass Campaign for Reactive Oral Cholera Vaccination in Rural Haiti

**DOI:** 10.1371/journal.pntd.0003050

**Published:** 2014-07-31

**Authors:** Jessica E. Teng, Dana R. Thomson, Jonathan S. Lascher, Max Raymond, Louise C. Ivers

**Affiliations:** 1 Division of Global Health Equity, Brigham and Women's Hospital, Boston, Massachusetts, United States of America; 2 Partners In Health, Boston, Massachusetts, United States of America; 3 Department of Global Health and Social Medicine, Harvard Medical School, Boston, Massachusetts, United States of America; 4 Zanmi Lasante, St. Marc, Haiti; University of California, Los Angeles, United States of America

## Abstract

**Background:**

In mass vaccination campaigns, large volumes of data must be managed efficiently and accurately. In a reactive oral cholera vaccination (OCV) campaign in rural Haiti during an ongoing epidemic, we used a mobile health (mHealth) system to manage data on 50,000 participants in two isolated communities.

**Methods:**

Data were collected using 7-inch tablets. Teams pre-registered and distributed vaccine cards with unique barcodes to vaccine-eligible residents during a census in February 2012. First stored on devices, data were uploaded nightly via Wi-fi to a web-hosted database. During the vaccination campaign between April and June 2012, residents presented their cards at vaccination posts and their barcodes were scanned. Vaccinee data from the census were pre-loaded on tablets to autopopulate the electronic form. Nightly analysis of the day's community coverage informed the following day's vaccination strategy. We generated case-finding reports allowing us to identify those who had not yet been vaccinated.

**Results:**

During 40 days of vaccination, we collected approximately 1.9 million pieces of data. A total of 45,417 people received at least one OCV dose; of those, 90.8% were documented to have received 2 doses. Though mHealth required up-front financial investment and training, it reduced the need for paper registries and manual data entry, which would have been costly, time-consuming, and is known to increase error. Using Global Positioning System coordinates, we mapped vaccine posts, population size, and vaccine coverage to understand the reach of the campaign. The hardware and software were usable by high school-educated staff.

**Conclusion:**

The use of mHealth technology in an OCV campaign in rural Haiti allowed timely creation of an electronic registry with population-level census data, and a targeted vaccination strategy in a dispersed rural population receiving a two-dose vaccine regimen. The use of mHealth should be strongly considered in mass vaccination campaigns in future initiatives.

## Author Summary

The World Health Organization (WHO) recently endorsed the creation of a global oral cholera vaccine (OCV) stockpile as part of an integrated, strategic framework to address the re-emerging threat that cholera causes worldwide. In conjunction, the WHO also called for continued monitoring and evaluation around the use of OCV in different settings. In response to the cholera epidemic in Haiti that began in October 2010, Partners In Health, an implementing partner of Haiti's Ministry of Health, vaccinated 50,000 Haitians in two rural communities in the Artibonite Valley in 2012. In this paper, the authors describe the use of mobile health (mHealth) technology for data collection and geospatial mapping to document this rural OCV campaign, focusing on the utility, benefits, and challenges of mHealth in a reactive campaign in the midst of the ongoing epidemic.

## Introduction

In mass vaccination campaigns and other large-scale health interventions, healthcare providers are challenged to find a data collection system that allows timely, accurate, and efficient management of large volumes of data. Traditional paper-based data collection systems have been used effectively in health programs for generations, and can result in high quality data. However, public health programs that use paper-based data systems and wish to analyze data must compensate for staff, paper records, time, and funding to digitize data, potentially adding vulnerability to human-introduced error [1]–[3]. With continuously expanding mobile networks [4], [5], mobile health (mHealth) solutions are an increasingly attractive and acceptable way to collect, manage, and analyze information [6], [7]. Direct electronic data collection can result in higher quality data [3], [8]–[13] and can produce a cleaner database more rapidly [3], [8], [10], [14], [15]. Direct data entry has also been shown to be faster and less expensive when compared to paper-based data collection [3]. Though employed in various health and research settings, the use of mHealth has not been extensively documented in mass vaccination campaigns in resource-limited settings.

The worst cholera outbreak in recent history has been ongoing in Haiti since October 2010, which began only ten months after a devastating earthquake struck near Port-au-Prince, the country's capital. As of October 2013, the Haitian Ministry of Health has reported over 8,360 cholera-related deaths and over 684,000 cases of cholera [16]. In December 2011, Haiti's Ministry of Health, and implementing partners Groupe Haïtien d'Étude du Sarcome de Kaposi et des Infectieuses Opportunistes (GHESKIO) and Partners In Health, initiated a plan to vaccinate 100,000 residents with oral cholera vaccine in two areas—50,000 in Port-au-Prince, and 50,000 in two rural communities in the Artibonite Valley. The context, strategies used, acceptability, feasibility, and vaccine coverage rates of these oral cholera vaccination (OCV) campaigns are described in detail elsewhere [17].

Fueled by an ongoing epidemic that continued to claim lives, there was urgency to complete vaccination prior to the forthcoming rainy season. We sought to demonstrate that a mass OCV campaign was possible in an epidemic, rural, resource-limited setting [18]. As a result, we documented individual-level information with greater detail than is typical in mass vaccination campaigns to understand granular-level coverage, uptake of the vaccine, trace vaccination adverse reactions, and evaluate vaccine effectiveness after the campaign [19]. We also wanted to ensure as complete vaccination coverage as possible to leverage the herd-immunity effect of the vaccine [20]–[22]. In order to collect the depth of information needed within a short period of time, we used an electronic mHealth solution with the primary aims of (1) providing a timely, efficient data management system, and (2) mapping and documenting detailed information on campaign community coverage and vaccine uptake for 50,000 vaccinees in two isolated communities in rural Haiti.

## Methods

### Ethics Statement

Ethics approval was obtained from Partners Institutional Review Board (Boston, MA) for secondary analysis of the data collected during the census and campaign. All data were collected as part of a public health campaign (approved by the Haitian National Bioethics Committee); informed consent was not required during the campaign.

### Study Area and Project Design

The vaccination campaign was conducted in Bocozel and Grand Saline, two sections in the Artibonite Valley of Haiti [17]. Bocozel was initially the targeted section for the campaign, but after census showed fewer than expected residents, the vaccination campaign area was extended to Grand Saline. Both of these agricultural communities are isolated and have poor road infrastructure, making access and travel to and within the areas difficult.

A census was conducted in Bocozel in February 2012 to pre-register eligible residents and to deliver an education campaign on hygiene and sanitation messages [17], [23]. All non-pregnant residents over 1 year old living in the section were invited to be vaccinated. A unique number was assigned to each house within each locality, and was marked on the door or wall of the house and recorded in the census database. Other variables recorded at the time of census were full name, gender, age, and locality (neighborhood) of residence. A census was not conducted in Grand Saline.

The vaccination campaign followed from April to June 2012 in two phases to avoid a schedule conflict with an oral polio vaccination campaign. Phase 1 targeted adults and children aged 10 years and older from Bocozel, and Phase 2 targeted children 1–9 years old from Bocozel, as well as all Grand Saline residents [17]. A three-tiered campaign strategy was used: community centers, schools, churches, and communal areas throughout the area were designated as fixed vaccination posts—44 in Bocozel and 11 in Grand Saline. When data demonstrated that attendance at the fixed vaccination posts began to wane, the supervisor and project managers moved to mobile posts, and subsequently to a door-to-door strategy.

### Equipment and Data Security

Data were collected using handheld Samsung (Seoul, South Korea) Galaxy Tabs with a 7-inch display running Android operating system; 50 tablets were used during the census, and 40 during the vaccination campaign. The software platform was built by a contracted partner (Majella Global Technologies, Portland, ME, USA) on Open Data Kit. External battery packs with dual USB charging ports provided a portable, backup power supply. Data records were first stored locally on devices in the field, and uploaded nightly via office Wi-Fi to a secure, web-hosted database. Online registries were subsequently downloaded nightly for analysis. All tablets and computers were password-protected and encrypted [24], [25].

### Barcodes and Vaccine Cards

Those interested in being vaccinated received a vaccine card either during the census, or at the vaccination post if they had not been included in the census for some reason. The vaccine card included a unique numeric barcode, full name, gender, age, locality (neighborhood) of residence, and spaces to record the date of each vaccination. We retrieved and input resident information by using the tablets' barcode-scanning function. Manual entry of the barcode number was possible if scanning failed. Receipt of each of two OCV doses was confirmed on the card with custom-made rubber stamps.

### Staff and Training

Locally recruited Haitians staffed 50 teams during the census and 40 teams during the vaccination campaign. Census teams of two had one enumerator and one community guide, and were managed by a total of 10 supervisors; vaccination teams consisted of one enumerator, two vaccinators, and one community guide, and were managed by a total of 20 supervisors. All enumerators were high school-educated.

Two-day trainings were conducted prior to the census and the vaccination campaign, covering use of hardware and software, in-depth review of the data collection forms, communication style, and role-play. We also conducted refresher trainings before each new dose throughout the campaign to review software functions, updates to data collection forms, feedback from the previous dose for improved data quality, and practice scenarios.

### Data Management

Each night, data records collected in the field were uploaded from each tablet and merged into a web-hosted database. At the end of census, population data were downloaded from the web-hosted electronic database and formatted in Microsoft Excel to become a dataset, or a “lookup table”. The lookup table was loaded back on to all tablets and embedded within the electronic forms, and served as a locally stored database from which previously collected population data could be retrieved. From April to June 2012, residents presented their cards at vaccination posts to receive OCV. Residents could go to any post, since census data was available on all tablets. Residents' barcodes were scanned and their personal census information automatically populated form fields (name, age, gender, locality of residence). We then added information at each encounter including enumerator and supervisor names, vaccination date, dose, and manufacturing batch number. The datasets were cleaned and newly updated lookup tables were loaded onto tablets between each vaccine phase.

**Figure 1:**
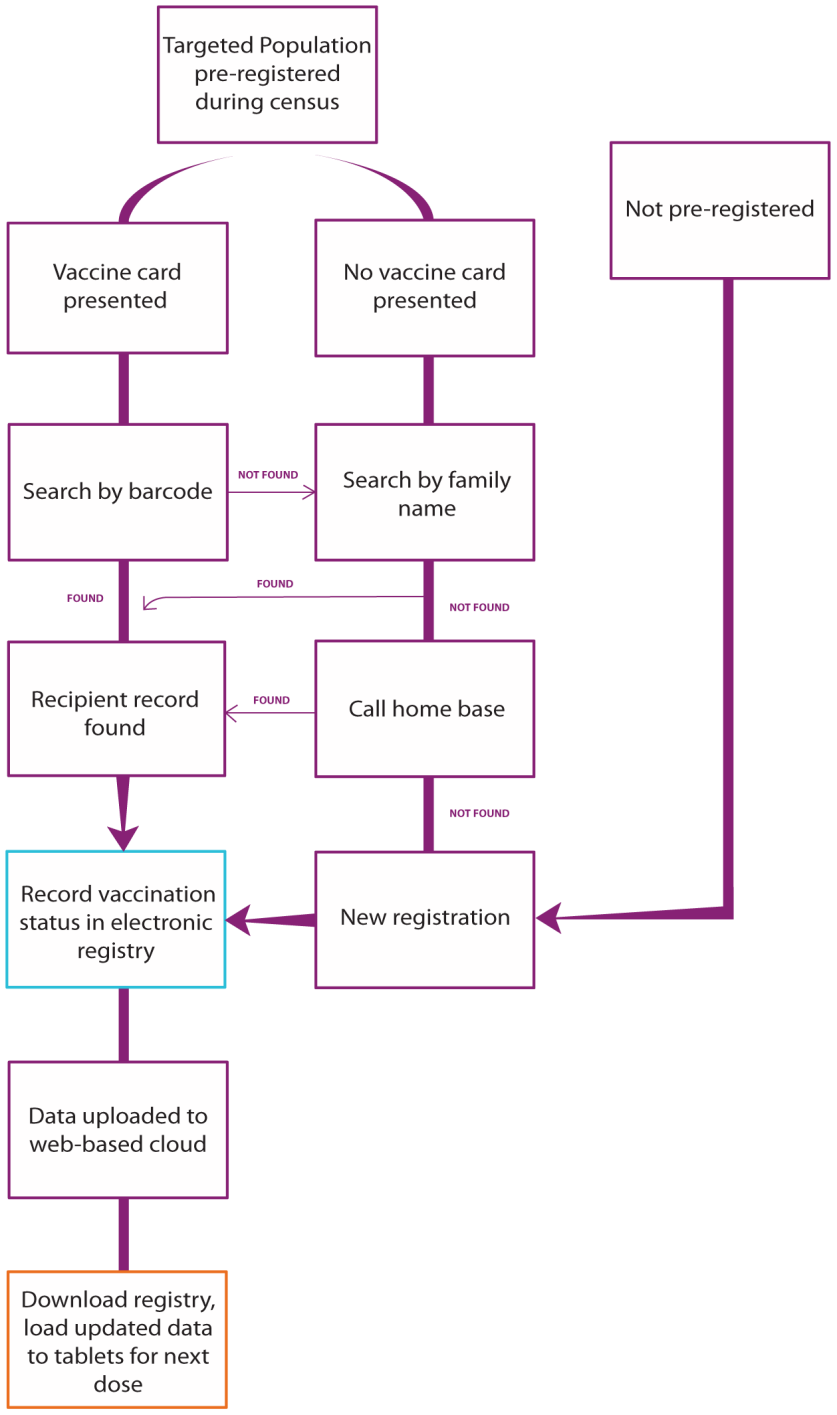
Flow diagram of mHealth vaccine registration for a reactive oral cholera vaccine campaign in rural Haiti, April–June 2012.

Each tablet had multiple data collection forms to provide appropriate ways to record information. The main two forms were autopopulated with vaccinee information by barcode, or by looking up the family name if a resident lost her/his vaccine card or if the barcode did not function. For those whose records could not be located on a tablet by barcode or family name, an office-based team could search any combination of identifiers in the database by desktop computer; we were then able to retrieve the original barcode number linked with the individual. If her/his record could not be located in the computer database, a new vaccine card was issued and a new registration form was completed to collect her/his information (see Figure 1).

After initial days of vaccination at fixed and mobile posts, we used our electronic databases and a custom-built analysis tool to generate case-finding reports of all residents who were expected to be vaccinated, but had still not received dose 1 (or who had received dose 1, but had not come for his/her second follow-up dose) [17]. For example, during phase 1, after vaccination records from the tablets were uploaded to the electronic registry database, we could compare the current vaccination registry with the original census database to identify individuals who had registered for OCV during the census, but still needed their first OCV dose. Our teams went door-to-door, locating individuals by the unique numbers assigned to each household during the census. There was no case-finding in Grand Saline because no census was performed there.

### Data Quality

The software included automatic check features, such as logic branching and requiring a response, to ensure accuracy and completeness before being saved. During data collection, team supervisors accompanied enumerators and did spot checks to ensure that enumerators were filling in forms correctly. If any errors were found, they were corrected on the tablet if possible, or else recorded in an error log and reported to the Data Manager for resolution in the electronic database daily or at the end of each phase. During vaccination, data collected on vaccine recipients for the vaccine registries were linked directly with census data in the tablet records, allowing for accurate data linking at the point of vaccination. The registries were reviewed nightly or every two nights, and cleaned at the end of each vaccination phase.

### Geospatial Mapping

**Figure 2:**
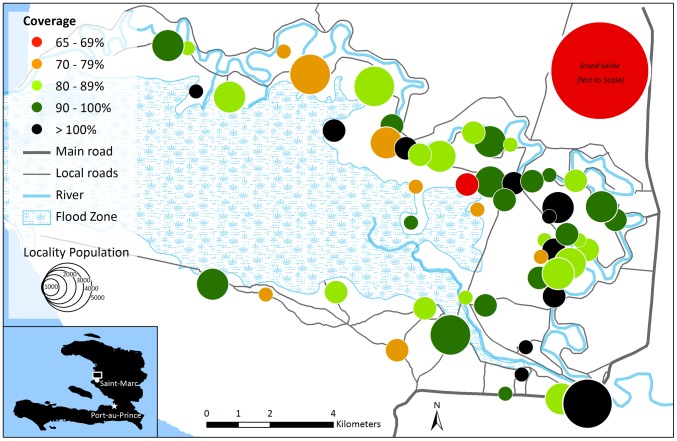
Map of community coverage in a reactive oral cholera vaccine campaign in rural Haiti, April–June 2012. Grand Saline, located directly north of the mapped area, is visualized to compare coverage rates to the rest of the campaign area, but its location and size are not to scale. Sub-area vaccination post locations were not collected in Grand Saline and therefore are not mapped.

**Figure 3:**
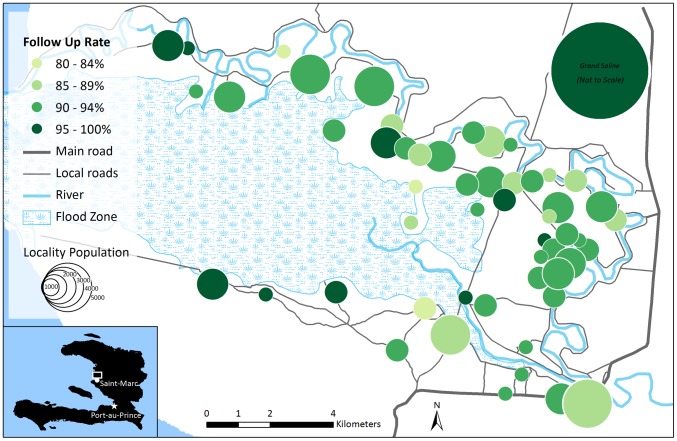
Map of vaccine follow-up rate of second doses in a reactive oral cholera vaccine campaign in rural Haiti, April–June 2012. Grand Saline, located directly north of the mapped area, is visualized to compare coverage rates to the rest of the campaign area, but its location and size are not to scale. Sub-area vaccination post locations were not collected in Grand Saline and therefore are not mapped.

Enumerators were trained how to take Global Positioning System (GPS) coordinates during staff training. Each morning of vaccination, they recorded the location of the fixed vaccination posts with the tablets' built-in GPS functionality. We mapped locality coordinates in Google Earth, verifying them with local collaborators and against existing databases [26]. We used ArcGIS 10 (ESRI, Redlands, California, USA) to compile this information and generate summary maps of localities, vaccination posts, regional health centers and cold chain storage locations, and population coverage rates. Because localities were recorded as point locations (rather than areas), we visualized community vaccination coverage (Figure 2) and follow-up rates (Figure 3) using population-proportionate bubbles centered over locality point locations. We shifted some locality points that were clustered together to reduce overlapping of the population bubbles and to improve visualization of the data. Additional roads, canals, and water bodies were geocoded from Google Earth to produce a background reference layer. Locality-level vaccination coverage data was not available in Grand Saline because we did not conduct a census there, so aggregated data were visualized.

### Cost of mHealth

The costs of hardware, software, and training were included in the calculations for determining the cost of implementing mHealth in the census and vaccination campaign in Bocozel and Grand Saline. Hardware costs included tablet computer purchase and rentals, and surge protectors. The cost of external battery packs and tablet accessories were excluded. Software costs included a data-hosting contract and mobile licenses for each tablet computer for the duration of the census and vaccination campaign, and the custom-built analysis tool for generating case-finding reports. Training costs included technology-related on-site training, support, and travel for two 1-week periods, which were estimated to be 40% of total training costs incurred by our contracted software platform partner.

## Results

During a 13-day census in Bocozel, we collected over one million pieces of data. Our teams enumerated an average of 14.6 households, or 53 individuals, per enumerator per day. We enumerated 34,767 individuals in 9,517 households. Of these, 33,441 eligible individuals pre-registered for OCV.

During 40 vaccination campaign days in Bocozel and Grand Saline, we collected approximately 1.9 million pieces of data while distributing 86,659 vaccines to 45,417 individuals. On the first vaccination day, when our vaccination posts were busiest, we documented administering OCV to 6,200 individuals, or 155 vaccinations per enumerator per day. Of those who received at least one dose during the campaign, 90.8% were also documented to receive two doses [17].

Case-finding reports were generated after initial days vaccinating at fixed and mobile posts during all phases of vaccine administration in Bocozel. When attendance at vaccination posts slowed and vaccination coverage based on census information plateaued, we created a case-generating report with address information of over 11,000 residents (approximately 20% of the target population for the campaign) of Bocozel who had registered for OCV, but had not yet received OCV.

Following the vaccination campaign, we mapped 55 vaccination posts and 4 health centers used as cold chain staging areas. We also mapped 53 localities in Bocozel, plus an additional point for Grand Saline. These data were used to generate maps of vaccination post locations [17], community coverage (Figure 2), and second-dose follow up (Figure 3).

The total cost of deploying mHealth during the campaign (including census in Bocozel, and vaccination in Bocozel and Grand Saline) was $29,129. Technology-related training cost $6,624; software and development totaled $7,900. Hardware purchase/rentals and surge protectors totaled $14,605, but the cost for hardware is flexible depending on negotiations, demand, and the regularly changing cost of technology.

## Discussion

This project demonstrates that mHealth is feasible and affordable in rural Haiti during a mass vaccination campaign. Direct entry electronic data gave us an iterative feedback capability, allowing us to track the progress of specific teams, and to give individualized feedback to staff as necessary. Nightly review of community coverage aided in determining the following day's vaccination strategy, whether fixed, mobile, or door-to-door within each locality. We used the electronic data to rapidly identify residents who had pre-registered during the census but were still not vaccinated, and prepared case-finding reports for distribution among teams the following day.

Although our mHealth solution required up-front financial investment and training, it eliminated the need for mass printing, data clerks, and manual data entry, which take time, are costly, and increase the risk of error [3], [27]. Conducting a census first to know our target population, monitoring campaign progress daily, and deliberate case-finding allowed our coverage and follow-up rates to be higher than they may have been using a paper registry system.

Both the hardware and software were user-friendly enough for high school-educated staff, which is consistent with other groups' findings [8], [28]. This is essential in settings where access to education is limited and literacy levels are low; in Haiti, youth literacy rates are 70–74%, but secondary school participation rate is only 18–21% [29]. Previous familiarity with computers and other phone user interfaces was an advantage in learning to navigate the software. The software used, in addition to other proprietary and open access software platforms, is generalizable to other country settings, and could be tailored as a data collection platform for other vaccination campaigns and health programs. Despite being exposed to a wide array of natural elements, including heat, light glare, humidity, rains, and dust, the tablets functioned well in the field. Tablet battery life was estimated to last eight hours, and external battery packs were used occasionally in the field.

We expected that documentation would be the most time-consuming part of the vaccination process (rather than actually administering OCV, which can be done quickly), and sought to optimize that process. Scanning barcodes has been shown to be a superior method of data collection for patient registration and vaccine tracking compared to paper-based methods, and this project supports those findings [30], [31]. Using tablets and barcodes saved time during data retrieval at the point of vaccination, shortening each participant encounter and allowing us to reach a large number of vaccinees each day. The barcodes were effective in accurately linking records to the same patient across different points of contact. At one point, we realized that several barcodes were scanning incorrectly. This was rectified by implementing numeric entry parameters in the electronic form so that only a 5-digit numeric entry would be accepted. When looking up records from the database, scanning barcodes to autopopulate forms on tablets saved time compared to using paper records, which would have required enumerators to search through a massive registry of participant records. Since the entire registry was stored locally on all tablets in lookup tables, residents could present their vaccine card at any post to receive OCV, not only the post in their locality of residence. Therefore, duplication of vaccination could have been possible if a resident presented at multiple posts, but using the custom-made rubber stamp to mark receipt of OCV on the recipients' vaccine cards discouraged this scenario. Using tablets also eliminated the heavy logistical preparations and time that would have been needed to sort paper records, digitize, print, and disperse them to all teams in the field simultaneously.

The use of mHealth contributed to our ability to supervise field activities. Reviewing daily progress was essential for informing our strategic plan for both immediate and upcoming tasks. Because the data was digitized during collection, the rapid availability of an electronic database and the ability to monitor data quality in near real-time proved useful for us; similar findings have been reported in mass influenza vaccination in Canada and in OCV in Tanzania [8], [32]. We could see how many people were being vaccinated each day and could thereby decide whether to change from fixed to mobile or door-to-door posts within localities. We also used the cumulative proportion of eligible residents vaccinated as an indicator to determine when to move teams around the campaign area to focus on those localities with lower proportions of vaccinated residents compared to other localities. Four enumerators made repeated errors in their forms, and three collected data very slowly and therefore had fewer records per day than the average enumerator. Supervisors were alerted and helped their teams to improve efficiency and accuracy. Having this birds'-eye view of progress across all localities enabled to better forecast our projected timeline.

We were able to quickly map community coverage and follow-up rates, creating an effective medium for visualizing data and also gain a deeper understanding of the reach of the campaign at the locality level. Collection of vaccination post geographic data happened seamlessly along with normal data collection activities, and one GIS specialist with basic spatial data management skills was able to plot and visualize that data. Figure 2 shows heterogeneity of coverage by locality. In some Bocozel localities, coverage was over 100% according to census counts. This was most likely attributable to the participation of non-residents, who presented on the day of vaccination without a vaccine card and claimed to live in Bocozel, but actually came from outside of the targeted area. Enumerators were trained to ask open-ended and follow-up questions about residency, but proof of residency in rural Haiti is almost impossible to document formally, and was not required for vaccination. In this way, the vaccination of non-residents likely contributed to the numerator without being included in the denominator in the census. Misclassified localities could also contribute to over-coverage. Localities of residence entered during vaccination phases overrode localities reported during the census in cases of discordant resident reporting. Five enumerators reported misclassifying locality for multiple consecutive records because of a ‘remember answer’ function in the tablet software that allowed a newly created record to automatically enter the same answer used in the previous record. Reported misclassifications were corrected in the final registries, but it is possible that unreported or unknown misclassifications remained in the registries. Finally, another possibility is that not all residents of Bocozel were reached and counted during the census, though this is unlikely.

The use of mHealth was particularly useful in this two-dose OCV campaign. In the setting of a cholera epidemic and humanitarian disaster, direct data entry allowed us to link vaccinee data across multiple points of contact, and monitor first dose coverage and second dose completion rates. Having the lookup tables on all tablets allowed vaccinees to receive their vaccine doses at any vaccination post, increasing flexible access to OCV. Other vaccine campaigns, whether single- or multiple- dose, may not require as close monitoring as is the case for this reactive OCV campaign during an epidemic, and in such cases, mHealth may not be as advantageous compared to a paper-based registry. Still, electronic data collection would be helpful in a single-dose vaccination campaign if implementers wanted to have an electronic dataset available shortly after the campaign, were interested in real-time progress monitoring, and had plans for geospatial data analysis.

Electronic data collection had limitations. Uploading records from the tablets to the web-hosted database was time-consuming. Although data clerks and administrative personnel were not needed to enter data and manage paper registries, we required personnel to manage our inventory of tablets and external batteries daily. Our goal of having real-time data was not always possible due to disrupted Wi-Fi in rural Haiti. Uploading records took 2–4 hours on average, and on nights without a strong Wi-Fi connection, took over 6 hours to complete. On rare occasion, uploading was incomplete before sending tablets back into the field the next day. For the same reason, we were unable to download and analyze the most updated registry on a couple occasions. Still, we were able to see data daily or every two days, as opposed to between phases or at the end of the campaign, which would have been the case with paper data collection. Despite improved data quality over manual entry and rapid availability, data cleaning was still necessary. On occasion, enumerators would report multiple, systematic errors made in the field that would have to be edited in the database. The web-hosted database was not an efficient interface for making data corrections, so these changes were recorded in a log and incorporated in the final database in Excel. Late in the campaign, we experienced some technical difficulties with the software on some tablets. We swapped out malfunctioning tablets until troubleshooting with developers resolved this issue.

Based on our experience during this pilot OCV campaign in rural Haiti, we have learned lessons for alternate solutions to the challenges we faced. Using mobile devices with SIM cards and cellular data plans would enable real-time syncing of data between the field and the central database. If data transfer speeds were capable of managing the data volume, this would streamline data-syncing by eliminating the time and personnel required to connect and sync numerous tablet computers on a nightly basis. This would also eliminate dependence on office-based Wi-fi that may not always be reliable. With SIM cards and cellular data connectivity in the field, lookup tables that served as locally stored databases on tablets would not have to be loaded on all tablets between multiple phases of vaccination, which took a considerable amount of time. Using real-time data connection, previously collected data (from the census, or dose 1) could be extracted directly from a web-hosted database and linked with newly collected data (from dose 1, or, 2, respectively). Another data-syncing solution would be to use secure digital cards to manually move records from mobile devices to one centralized database stored locally on an office-based server; this method may require additional personnel, and we did not have the option of a local server for our campaign. While direct data entry can eliminate the need for data entry and validation, it is important not to underestimate the staffing needs for data and mobile device management and technical maintenance on a daily basis. Because mHealth system provides access to additional information on the progress of field teams, more time for individual feedback with enumerators could have resulted in higher quality data collection. Finally, while our tablet forms were written in Haitian Creole, the software interface itself was in English, and it would be advantageous to have all software instructions and options in the native language of our enumerators.

To our knowledge, there is only one paper describing in detail the benefits and use of mHealth during an OCV campaign [8]. The Zanzibar, Tanzania campaign targeted both urban and rural communities with high-risk populations in a pre-emptive public health vaccination campaign in a cholera-endemic setting. In contrast, our paper describes a reactive vaccination campaign during an ongoing cholera epidemic in a highly dispersed rural population in the midst of a complex emergency setting. Both pieces add to the literature on documenting OCV campaigns from which campaign results and summaries are rapidly accessible, which may also shorten the delay of subsequent disease surveillance activities. This is particularly relevant for current and upcoming OCV campaigns, which may become more frequent as a result of WHO's endorsement of a global OCV stockpile [33], [34] and evolving policy recommendations to vaccinate both in endemic regions and areas of outbreak [35]–[37]. Furthermore, these findings are generalizable beyond mass OCV campaigns to other types of epidemics. The capacity to vaccinate reactively with efficiency and accuracy, and also conduct surveillance post-vaccination, can be conferred to other mass vaccination programs in resource-limited settings to document vaccination coverage, do active case-finding, and potentially increase coverage and follow-up rates in the campaign.

### Conclusion

The use of mobile health technology in a reactive OCV campaign in Haiti allowed timely creation of an electronic vaccination registry with population-level census data, and a targeted vaccination strategy in a dispersed rural population receiving a two-dose vaccine regimen. Direct-entry electronic registries allowed us to evaluate community coverage in near real-time, and to generate case-finding reports that resulted in greater than had occurred with fixed vaccination posts. The collection of GPS coordinates for geospatial mapping allowed us to calculate, plot and visualize community coverage and vaccination follow-up rates, which were important programmatic results in the setting of an epidemic and humanitarian crisis. The use of mHealth should be strongly considered in future mass vaccination campaigns, including during epidemics and situations in which timely access to vaccination coverage data is required.
